# Polarization of concave domains by traveling wave pinning

**DOI:** 10.1371/journal.pone.0190372

**Published:** 2017-12-28

**Authors:** Slawomir Bialecki, Bogdan Kazmierczak, Tomasz Lipniacki

**Affiliations:** 1 Institute of Fundamental Technological Research, Polish Academy of Sciences, Warsaw, Poland; 2 Department of Statistics, Rice University, Houston, Texas, United States of America; Queensland University of Technology, AUSTRALIA

## Abstract

Pattern formation is one of the most fundamental yet puzzling phenomena in physics and biology. We propose that traveling front pinning into concave portions of the boundary of 3-dimensional domains can serve as a generic gradient-maintaining mechanism. Such a mechanism of domain polarization arises even for scalar bistable reaction-diffusion equations, and, depending on geometry, a number of stationary fronts may be formed leading to complex spatial patterns. The main advantage of the pinning mechanism, with respect to the Turing bifurcation, is that it allows for maintaining gradients in the specific regions of the domain. By linking the instant domain shape with the spatial pattern, the mechanism can be responsible for cellular polarization and differentiation.

## Introduction

Pattern formation is a ubiquitous phenomenon in biology and is observed at different scales [1, 2]. Spatial patterns arise in populations of living organisms [3–8], are crucial in morphogenesis [9–11], and are closely associated with intracellular signal transduction [12–15]. Spatial gradients of protein concentration can regulate the process of cell division [16] and motion [12, 17]. Cell polarity, ubiquitous for uni- and multicellular organisms, enables differential inheritance during cell divisions [18].

There are several mathematical theories proposing potential mechanisms of polarization and pattern formation [19]. The most recognized is the Turing bifurcation theory [20], in which spatially nonuniform solutions arise in systems with two or more reaction–diffusion equations with different diffusion coefficients [2, 21]. Oscillatory Turing-like patterns can arise also in fluids due to mechanochemical coupling [22]. The group of Edelstein-Keshet proposed an interesting mechanism in which traveling front in a bistable system decelerates and becomes stationary due to the global depletion of a fast diffusing variable as a consequence of front propagation [23]. This mechanism also requires (at least) two equations with different diffusion coefficients. Another similar mechanism known as local excitation/global inhibition (LEGI), that arises in spatial systems exhibiting relaxation oscillations, provides spatial patterns fluctuating in time [17]. Domain polarization can arise also in systems in which two opposing processes (e.g., phosphorylation and dephosphorylation) occur in different subcompartments [14, 15, 24, 25].

Here, we explore a different mechanism, which can lead to polarization of three-dimensional concave domains by pinning of traveling fronts. This mechanism requires only a single reaction–diffusion equation

$$\begin{array}{c} \frac{\partial u}{\partial t}=D\nabla^{2}u+f\left(u\right), \end{array}$$ (1)

where *D* is the diffusion coefficient, with bistable source function *f*(*u*) supplemented by the non-flux boundary condition. In 1978, Casten and Holland showed that Eq (1) with homogeneous Neumann boundary conditions has no stable nonuniform equilibrium solutions provided that the domain *Ω* is convex [26]. Later, Matano showed that the assumption of convexity is crucial [27]. Recently, it has been proved that in cylindrical domains with abrupt opening, traveling wave may be blocked [28].

We will give the criteria for the position of the pinned front and its stability in axisymmetric domains and verify accuracy of these criteria numerically. Next, for various concave domains (in general, not axisymmetric), we give examples of different types of nonuniform solutions in volumes obtained via the front pinning phenomenon, associated with the analyzed mechanism. Importantly, although our theoretical analysis becomes strict only in the limit of very small diffusivities, such nonuniform solutions remain robust also for large diffusion coefficients, when front thickness is comparable with the size of the domain.

## Results

### Traveling front velocity and stationary solutions

We will focus on Eq (1), and assume that *f*(*u*) is bistable, with stable steady states *u*_−_ and *u*_+_, and an unstable steady state *u*_0_ ∈ (*u*_−_, *u*_+_). We will analyze solutions to Eq (1) in compact 3D domains with nonflux boundary conditions. In Eq (1) the operator ∇^2^(⋅) = ∇ ⋅ ∇(⋅) is the classical Laplace operator in 3D.

In three dimensions, the local front velocity $\vec{V}$, i.e., the local velocity of the surface *f*(*u*) = 0, is given by the eikonal equation [29]

$$\begin{array}{c} \vec{V}=\vec{c}+D\left(\vec{\kappa_{1}}+\vec{\kappa_{2}}\right), \end{array}$$ (2)

where $\vec{c}$, $\vec{\kappa_{1}}$, and $\vec{\kappa_{2}}$ are vectors perpendicular to the surface *f*(*u*) = 0, with lengths equal, respectively, to the front velocity in 1D, and local principal curvatures of the front surface. The vectors $\vec{\kappa_{1}}$ and $\vec{\kappa_{2}}$ are directed towards the center of the strictly tangent ellipsoid. Eq (2) is a good approximation in the limit of *D* → 0, i.e., when the front thickness, proportional to $\sqrt{D}$, is much smaller than the inverses of $|{\vec{\kappa }}_{1}|$ and $|{\vec{\kappa }}_{2}|$. When *c* = 0, front moves in such a direction that front surface shrinks. When *c* ≠ 0, the curvature term may have either the same or the opposite sign to *c*. Eq (2) implies that a spherical front is stationary, when its radius of curvature is *R* = 2*D*/*c*. Such a stationary solution is unstable since any increase of its curvature radius will result in the further front expansion, whereas any decrease of *R* will result in further front shrinking. However, as we will see below in bounded concave domains, there may exist stable stationary fronts.

**Fig 1:**
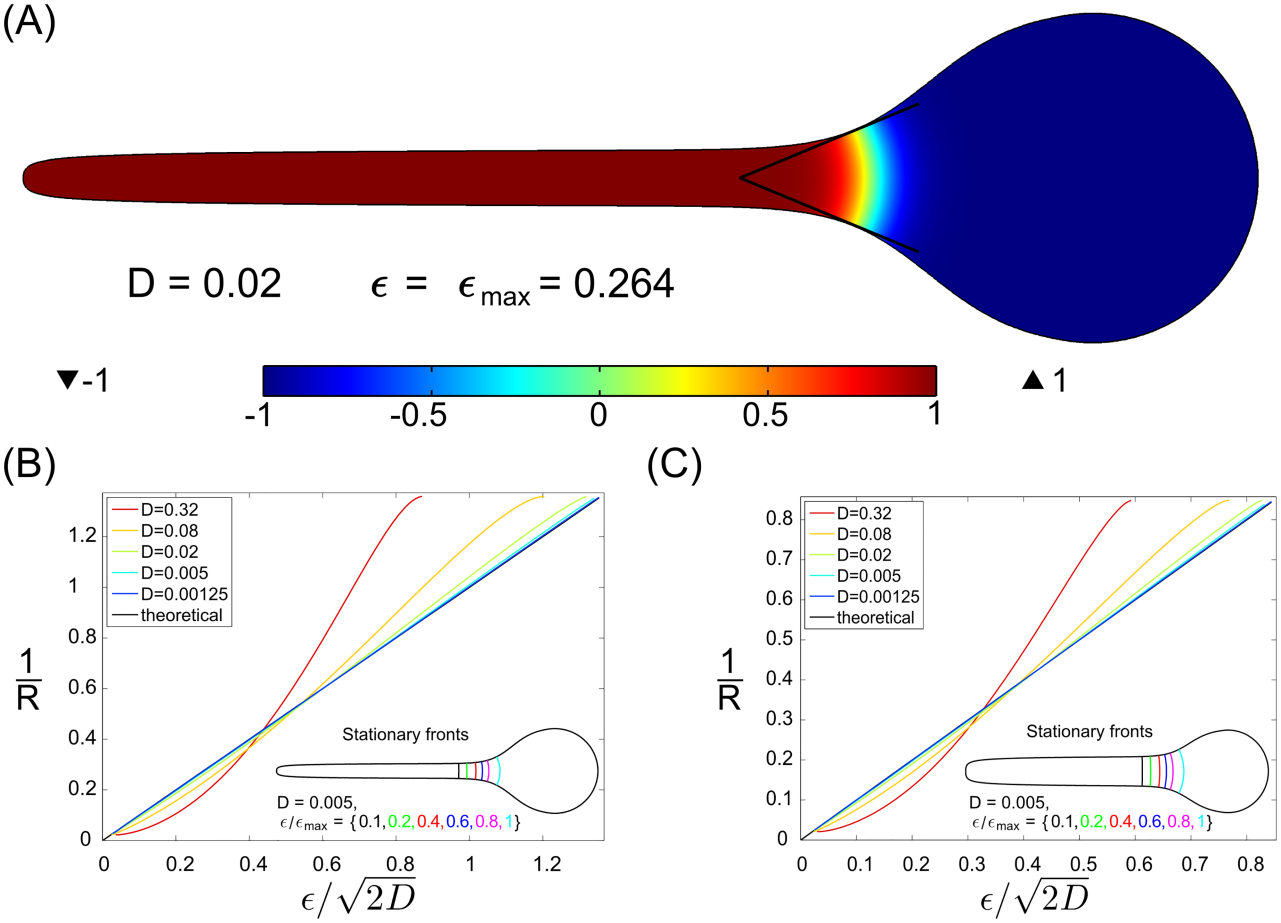
Fronts pinned in the local widening of the 3D cylindrical domain. The width of the simulation domain is 2. (A) An example stable stationary solution for *D* = 0.02 and *f*(*u*) = (1 − *u*)(*u* + 1)(*u* + *ϵ*), *ϵ* = *ϵ*_*max*_ = 0.264. Cross-section plane containing axis of symmetry is shown. The surface *f*(*u*) = 0 defines the position of the stationary front. (B-C) the curvature *κ* = 1/*R* of the stationary fronts calculated from numerical simulations for *f*(*u*) = (1 − *u*)(*u* + 1)(*u* + *ϵ*), in the domain with the diameter ratio 0.2 (B) and 0.4 (C) for five values of *D*: 0.00125, 0.005, 0.02, 0.08, 0.32 (colors from dark blue to red) versus the analytical result given by Eq (8) (black line overlapping with dark blue line) in the *D* → 0 limit. The front surface position is determined by its radius of curvature via Eq (3). Inserts in (B) and (C) show cross-section of the stationary front surfaces with the cylinder symmetry plane for *D* = 0.005 and six values of *ϵ*/*ϵ*_max_: 0.1, 0.2, 0.4, 0.6, 0.8, 1; *ϵ*_max_ = 0.134 for diameter ratio 0.2 (B) and *ϵ*_max_ = 0.0832 for diameter ratio 0.4 (C).

To investigate such stable fronts, let us consider an axially symmetric cylinder bounded by the surface *r*(*ϕ*, *z*) = *r*(*z*), where *ϕ*, *z* are cylindrical coordinates. Approximating locally the cylinder by the tangent cone (Fig 1A), we find, by the symmetry, that the front is spherical, and locally perpendicular to the cylinder boundary. Therefore its radius of curvature equals

$$\begin{array}{c} R\left(z\right)=r\sqrt{1+{\left(\frac{dr}{dz}\right)}^{-2}}, \end{array}$$ (3)

where the derivative *dr*/*dz* is calculated at *z* at which front surface intersects with the cylinder surface. Let us assume that *c* is positive, i.e., that for *r* = const the front propagates in the direction of increasing *z*, and that *dr*/*dz* > 0 as in the region in Fig 1A where the stationary front localizes. Then the front velocity is

$$\begin{array}{c} V\left(z\right)=c-\frac{2D}{R(z)}. \end{array}$$ (4)

It follows that if for some *z* = *z*_0_

$$\begin{array}{c} \frac{2D}{R(z_{0})}=c, \end{array}$$ (5)

then there exists a stationary solution to Eq (1). If additionally

$$\begin{array}{c} (\frac{dR}{dz}){|}_{z=z_{0}}>0, \end{array}$$ (6)

then *V*(*z*) > 0 for *z* < *z*_0_ and *V*(*z*) < 0 for *z* > *z*_0_, thus this stationary solution is stable. For *dr*/*dz* ≪ 1, radius of front curvature given by Eq (3) can be approximated as *R* ≈ *r*(*dr*/*dz*)^−1^, but such approximation is imprecise for abrupt cylinder openings (such as those shown in Fig 1A), and in the further analysis we will use Eq (3). For numerical verification of accuracy of the pinned front radius estimate we take

$$\begin{array}{c} f(u)=(1-u)(1+u)(u+\epsilon ). \end{array}$$ (7)

Thus *f*(*u*) is bistable, with stable steady states *u*_−_ = −1, *u*_+_ = 1, and an unstable steady state *u*_0_ = −*ϵ* ∈ (−1, 1). This choice of *u*_−_, *u*_+_ does not decrease the generality of formula (7), because any third order reaction term with two stable steady states can be transformed to the above case by a linear change of variables. For such *f*(*u*), the traveling front velocity in 1D is $c=\epsilon \sqrt{2D}$ (see, e.g. [1]) which inserted to Eq (5) gives the curvature of the pinned front

$$\begin{array}{c} \kappa =\frac{1}{R}=\frac{\epsilon }{\sqrt{2D}}. \end{array}$$ (8)

The above formula is verified in numerical simulations in two geometries with different ratios of the radii of the cylindrical to spherical part of the domain (Fig 1B and 1C). The simulations were performed in COMSOL using the finite-element method, see Methods for details. Radius of a pinned front determines implicitly by formula (3) its *z*-position, and this formula was used to determine *R* from numerical simulations. The stationary fronts localize in the region *dR*/*dz* > 0 (in agreement with Eq (6)), and for higher values of *ϵ*, the fronts pin at points *z* at which their curvature *κ*(*z*) = 1/*R*(*z*) is higher (in agreement with Eq (4)). For a given geometry and the diffusion coefficient *D*, there exists a critical, maximum value of *ϵ* = *ϵ*_*max*_, above which fronts may not be pinned at the boundary.

For *ϵ* ≪ 1, i.e., when the radius of curvature of the pinned front *R* is much smaller than front thickness equal $\sqrt{2D}$ (see [1]), the analytical formula (8) holds with a perfect accuracy. It is worth noticing that the stationary solution shown in Fig 1A was obtained for *ϵ* = 0.2 and *D* = 0.02. For such *D* the front thickness $\sqrt{2D}=0.2$ is comparable with its radius of curvature $R=\epsilon /\sqrt{2D}=1$, but still the formula (8) well approximates the numerical solution (Fig 1B).

### Domain polarization by traveling wave pinning

**Fig 2:**
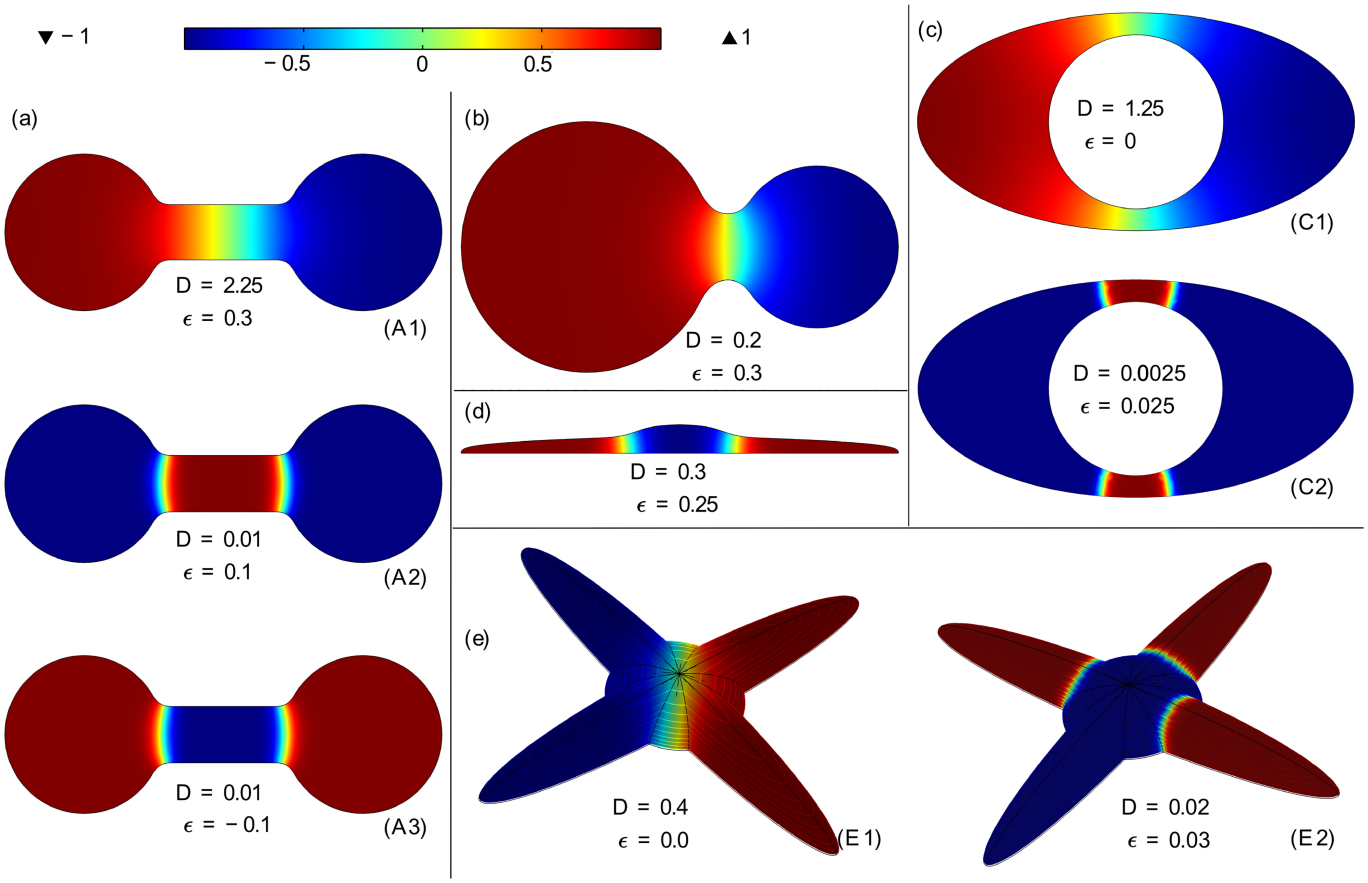
Stable nonuniform solutions in different 3D concave domains. (A) Symmetric dumbbell. Two classes of stable nonuniform solutions exist either with one ((A1)), or two ((A2) and (A3)), stationary fronts. Solutions of type (A1) exist for |*ϵ*| < *ϵ*_a1_(*D*) > 0. Solutions of type (A2) exist for 0 < *ϵ*_a2_(*D*) < *ϵ* < *ϵ*_a3_(*D*), while solutions of type (A3) exist for −*ϵ*_a3_ < *ϵ* < −*ϵ*_a2_. (B) Asymmetric dumbbell. Stable nonuniform solutions with *u* = 1 in the larger bell exist for 0 > −*ϵ*_b1_(*D*) < *ϵ* < *ϵ*_b2_(*D*) > 0, while analogous solutions with *u* = 1 in the smaller bell exist for −*ϵ*_b2_(*D*) < *ϵ* < *ϵ*_b1_(*D*). (C) Ellipsoidal cell with spherical nucleus and nonflux boundary conditions on outer and inner boundaries. Solutions of type (C1) exist for |*ϵ*| < *ϵ*_c1_(*D*) > 0, while solutions of type (C2) exist for $0<{\underline{\epsilon }}_{\text{c2}}\left(D\right)<\epsilon <{\bar{\epsilon }}_{\text{c2}}\left(D\right)>0$. (D) “Adherent cell”. Stable solutions with one or two standing fronts exist for $0<{\underline{\epsilon }}_{\text{d1}}\left(D\right)<\epsilon <{\bar{\epsilon }}_{\text{d1}}\left(D\right)>0$. (E) Symmetric “adherent cell” with four protrusions. Solutions with a single front of type (E1) exist for |*ϵ*| < *ϵ*_e1_(*D*) > 0. Solutions with one, two, three or four standing fronts of type (E2) exist for $0<{\underline{\epsilon }}_{\text{e2}}\left(D\right)<\epsilon <{\bar{\epsilon }}_{\text{e2}}\left(D\right)>0$. The values of ${\underline{\epsilon }}_{\text{e2}}$ and ${\bar{\epsilon }}_{\text{e2}}$ depend on the number of fronts and their configuration.

Pinning of traveling fronts can lead to 3D domain polarization, or to formation of more complex patterns when the number of pinned fronts is larger than 1. In Fig 2 we give several examples of stable patterns that can be formed in 3D domains resembling living cells or other biological objects, like embryos or bones. It is worth noticing that even for domains of relatively simple shapes such as a symmetric dumbbell (Fig 2A), or an ellipsoidal cell with spherical nucleus (Fig 2C), there can exist more than one stationary front, giving rise to several topologically nonequivalent solutions. For the domain considered in Fig 2E, chosen to resemble cell with four protrusions, there can exist simultaneously up to four stationary fronts, and therefore the stationary solution can approach *u*_+_ = 1 or *u*_−_ = −1 in arbitrary combination of protrusions. For a given domain the specific configuration of stationary fronts can exist in some range of parameters *ϵ* and *D*, as specified in Fig 2 caption. For geometries having local narrowing (like dumbbell), the nonuniform solutions are robust, i.e., they exist for a broad range of *ϵ* and *D*.

## Discussion

It is common that stable stationary solutions to equations of mathematical physics correspond to the minima of some potential. Let us notice that in our case, the stationary counterpart of (1),

$$\begin{array}{c} D\nabla^{2}u-\frac{\partial V}{\partial u}=0, \end{array}$$ (9)

can be derived as the Euler–Lagrange equation corresponding to the energy functional

$$\begin{array}{c} E\left(u\right):=\int_{\Omega }(V\left(u\left(x\right)\right)+\frac{1}{2}D{\left|\nabla \;u\left(x\right)\right|}^{2})dx, \end{array}$$ (10)

where

$$\begin{array}{c} V\left(u\right)=-\int_{\left(\cdot \right)}^{u}f\left(s\right)ds. \end{array}$$ (11)

The potential *V*(*u*) has two minima at *u*_−_ and *u*_+_, and one maximum at *u*_0_; in these points *f*(*u*) = 0. For the traveling wave solutions *u*(*z*, *t*) = *u*(*z* − *ct*), the function *u*(⋅) passes through point *u*_0_, which implies that the portion of the domain in the vicinity of the traveling front surface significantly contributes to the energy functional (10). In cylinders with a constant cross-section, traveling fronts propagate in the way that the region in which the solution approaches the stable steady state of the lower energy expands. In irregular domains, however, propagating fronts typically undergo shrinkage or elongation, which changes the front-associated energy. The traveling front can be thus pinned at a position that minimizes energy functional (10). In the case when potential *V*(*u*) has equal minima (corresponding in our case to *ϵ* = 0), the front pins in the position in which its surface area attains a local minimum. Here, we propose the term ‘pinning’, by analogy to quantum vortices which also tend to minimize their length-associated energy and thus pin to boundary protrusions [30].

The main advantage of the proposed polarization mechanism is that it links the instant shape of the domain with the arising pattern. The stable stationary fronts can be formed in 3D concave domains. In axisymmetric domains, pinned fronts are by the symmetry spherical. In more complex geometries, front surface is determined by the eikonal equation

$$\begin{array}{c} \kappa_{1}+\kappa_{2}=\frac{c}{D}, \end{array}$$ (12)

i.e., has a constant mean curvature. The discussed solutions, in contrast to the nonuniform solutions provided by the Turing bifurcation [1] and those obtained within the Edelstein-Keshet model [23], exist even for a single scalar bistable reaction–diffusion equation. In a PDE system however, other equations, even if monostable per se, when coupled to the gradient-providing equation can give birth to a complex spatial pattern.

The front pining mechanism can be responsible for establishing gradients before asymmetric cell divisions, allowing for cellular differentiation. As shown in Fig 2C, such gradients can be stable even in convex cells, in the case when there is no flux through the nuclear membrane. In unicellular organisms such as *Caulobacter crescentus*, *Bacillus subtilis* and *Saccharomyces cerevisiae*, prior to division, fate-dictating proteins preferentially segregate into one of the future daughter cells [31, 32]. Such type of polarization also enables cellular differentiation in multicellular organisms.

Polarization is also important for some types of non-dividing cells; it enables a developing neuron to convert one of its protrusions into axon, and the remaining ones into dendrites [33]. In this case, the front can be established in the opening called axon hilock, as in the solution shown in Fig 2E (subpanel E2). As demonstrated in [34], polarizing traveling fronts can arise also due to stochastic fluctuations, which occur more eagerly in narrow parts of the domain.

We focused on scalar bistable equations, but the same methodology can be applied also to the bistable systems satisfying monotonicity conditions [35]. The more challenging is the situation when a fast bistable equation is coupled to the slow ‘repression’ equation as in the FitzHugh-Nagumo model [36]. Depending on parameters, such systems can exhibit bistable, excitable or oscillatory traveling waves [37]. Based on our analysis, we may expect that in the excitable regime, when the system is monostable, traveling fronts unable to pass trough domain opening will vanish. Overall, our analysis shows that for bistable or excitable systems specific domain geometry can impose polarization or restrict propagation of excitable fronts.

## Methods

All numerical simulations in this study were performed in COMSOL Multiphysics 4.3b. The COMSOL codes that can be used to reproduce Figs 1A and 2 are provided in S1 File. COMSOL solves initial–boundary value reaction–diffusion equations by means of the finite-element method. The accuracy of calculations depends mainly on the finite-element mesh size, which should be carefully chosen. The mesh sizes were adjusted so that their further refinement does not lead to any visible changes in the result. COMSOL default values were used for ‘relative tolerance’ and ‘absolute tolerance’ parameters.

In numerical simulations for Fig 1 we benefit from the axial symmetry of the domain, which allowed us to reduce the problem from 3-dimensional to 2-dimensional using the Laplace operator in cylindrical coordinates, i.e.,

$$\begin{array}{c} \Delta =\frac{\partial^{2}}{\partial r^{2}}+\frac{1}{r}\frac{\partial }{\partial r}+\frac{\partial^{2}}{\partial z^{2}}. \end{array}$$ (13)

In this way, the calculations were confined to a half-plane {(*r*, *z*, *ϕ*): *ϕ* = 0, *r* ≥ 0} with the additional homogeneous Neumann boundary condition at the boundary *r* = 0 (coinciding with the *z*-axis).

The simulations performed for Fig 1 started from initial data in the form

$$\begin{array}{c} u_{t=0}\left(\phi ,r,z\right)=1-2H\left(z-z_{0}\right), \end{array}$$ (14)

where *H*(⋅) is the Heaviside step function, with *z*_0_ lying in the portion of the domain where the diameter of the cross-section (perpendicular to *z*-axis) is nearly constant. The traveling fronts move in +*z* direction and in each case we run the simulations until the front stops, i.e., the steady state solution is reached. Because of the adaptive time step, simulations converging to the steady state solutions are of low computational cost.

To obtain the results shown in Fig 1A we determine the value of *ϵ*_max_ using the bisection method. To recall, *ϵ*_max_ (defined in subsection Results) is the upper bound of *ϵ* for which a non-homogenous stationary solution exists. To obtain plots shown in Fig 1A and 1B, the radii of curvature of the pinned fronts, *R*, were calculated from front-pinning *z*-positions using Eq (3).

The two cylindrical domains considered in Fig 1 are bounded by the cylindrical surfaces *r*(*ϕ*, *z*) = *r*(*z*). The function *r*(*z*) was defined as

$$\begin{array}{c} r\left(z\right)=\frac{h_{1}\times p_{3}\times z^{1/2}}{p_{1}+p_{3}\times z^{1/2}}+\frac{\left(p_{4}-h_{1}\right)\times {\left(p_{5}\times z\right)}^{k}}{p_{2}+{\left(p_{5}\times z\right)}^{k}}\;\text{for}\;z\in \left[0,z_{1}\right] \end{array}$$ (15)

and

$$\begin{array}{c} r\left(z\right)=\sqrt{R^{2}-{\left(z-z_{s}\right)}^{2}}\;\text{for}\;z\in \left(z_{1},z_{s}+R\right] \end{array}$$ (16)

with

$$\begin{array}{ccc} z_{\text{s}} & = & z_{1}+{\text{lim}}_{z\to z_{1}^{\left(-\right)}}\left(r\left(z\right)\frac{dr}{dz}\left(z\right)\right),\\ R & = & {\text{lim}}_{z\to z_{1}^{\left(-\right)}}\left(r\left(z\right)\sqrt{1+{\left(\frac{dr}{dz}\left(z\right)\right)}^{2}}\right), \end{array}$$ (17)

where *z*_1_ = 6.3121. The function defined by Eq (16) is chosen so that both *r*(*z*) and $\frac{dr}{dz}$ are continuous at *z*_1_. The parameters *p*_1_ = 0.5, *p*_2_ = 2.0, *p*_3_ = 1.6784, *p*_4_ = 1.0650, *p*_5_ = 0.1878, *k* = 20 are common for domains considered in Fig 1B and 1C whereas *h*_1_ = 0.2 and *h*_1_ = 0.4 are specific for Fig 1B and 1C, respectively. The parameter values assure that the simulation domains have width equal to 2.0; this value sets the scale for the considered diffusion coefficients. Also, the characteristic dimension of domains considered in Fig 2 is 2.0. The stationary fronts localize within [0, *z*_1_], thus the function *r*(*z*) defined by Eq (16) for *z* > *z*_1_ plays only an auxiliary role.

To obtain the non-uniform stationary solutions shown in Fig 2 the same technique as that for Fig 1 has been applied. We started from the initial data in the form *u*_*t*=0_ = *u*_−_ = −1 or *u*_*t*=0_ = *u*_+_ = 1 in appropriate subregions of the considered domains and allow the evolution to proceed, until the solution reaches its stationary state. All simulations for Fig 2 were performed in 3D, even in the case when axial symmetry allows for reducing the problem to 2D. Of note, even in 3D the typical simulation time is of order of minutes on a standard PC.

## Supporting information

S1 File The COMSOL multiphysics binary files. In this zipped directory we provide COMSOL files to reproduce all results shown in Figs 1A and 2.
(ZIP)
